# Comparison of the transcriptome, lipidome, and c-di-GMP production between BCGΔBCG1419c and BCG, with Mincle- and Myd88-dependent induction of proinflammatory cytokines in murine macrophages

**DOI:** 10.1038/s41598-024-61815-8

**Published:** 2024-05-24

**Authors:** Mario Alberto Flores-Valdez, Eliza J. R. Peterson, Michel de Jesús Aceves-Sánchez, Nitin S. Baliga, Yasu S. Morita, Ian L. Sparks, Deepak Kumar Saini, Rahul Yadav, Roland Lang, Dulce Mata-Espinosa, Juan Carlos León-Contreras, Rogelio Hernández-Pando

**Affiliations:** 1 Biotecnología Médica y Farmacéutica, Centro de Investigación y Asistencia en Tecnología y Diseño del Estado de Jalisco, A.C., Av. Normalistas 800, Col. Colinas de la Normal, 44270 Guadalajara, Jalisco Mexico; 2 Institute for Systems Biology, Seattle, WA 98109 USA; 3 Department of Microbiology, University of Massachusetts, 639 N Pleasant St, Amherst, MA 01003 USA; 4 Department of Developmental Biology and Genetics, Indian Institute of Science, Bangalore, 560012 India; 5 Institut für Klinische Mikrobiologie, Immunologie und Hygiene, Universitätsklinikum Erlangen, Erlangen, Germany; 6 Instituto Nacional de Ciencias Médicas y Nutrición Salvador Zubirán, Vasco de Quiroga 15, Belisario Domínguez Sección 16, Tlalpan, Mexico City, Mexico

**Keywords:** BCG, Tuberculosis, RNASeq, Transcriptomic, c-di-GMP, Lipids, Cytokines, Bacterial genetics, Transcriptomics

## Abstract

We have previously reported the transcriptomic and lipidomic profile of the first-generation, hygromycin-resistant (Hyg^R^) version of the BCGΔBCG1419c vaccine candidate, under biofilm conditions. We recently constructed and characterized the efficacy, safety, whole genome sequence, and proteomic profile of a second-generation version of BCGΔBCG1419c, a strain lacking the *BCG1419c* gene and devoid of antibiotic markers. Here, we compared the antibiotic-less BCGΔBCG1419c with BCG. We assessed their colonial and ultrastructural morphology, biofilm, c-di-GMP production in vitro, as well as their transcriptomic and lipidomic profiles, including their capacity to activate macrophages via Mincle and Myd88. Our results show that BCGΔBCG1419c colonial and ultrastructural morphology, c-di-GMP, and biofilm production differed from parental BCG, whereas we found no significant changes in its lipidomic profile either in biofilm or planktonic growth conditions. Transcriptomic profiling suggests changes in BCGΔBCG1419c cell wall and showed reduced transcription of some members of the DosR, MtrA, and ArgR regulons. Finally, induction of TNF-α, IL-6 or G-CSF by bone-marrow derived macrophages infected with either BCGΔBCG1419c or BCG required Mincle and Myd88. Our results confirm that some differences already found to occur in Hyg^R^ BCGΔBCG1419c compared with BCG are maintained in the antibiotic-less version of this vaccine candidate except changes in production of PDIM. Comparison with previous characterizations conducted by OMICs show that some differences observed in BCGΔBCG1419c compared with BCG are maintained whereas others are dependent on the growth condition employed to culture them.

## Introduction

Tuberculosis (TB) is a transmissible, predominantly respiratory disease where cases and related mortality increased worldwide to an estimated 10.6 million cases with around 1.3 million associated deaths in 2022^1^. The only vaccine currently in use to prevent disseminated and miliary TB during childhood, *Mycobacterium bovis* Bacille Calmette-Guèrin (BCG) has diverse shortcomings^2^ that have led to development of several novel vaccine candidates, which are at different stages of preclinical and clinical characterization^3^ (https://newtbvaccines.org/tb-vaccine-pipeline/preclinical-stage/). Among the strategies intended to replace or improve BCG, there are novel, live, attenuated, mycobacteria-based vaccine (LAV) candidates, which aim to increase safety, immunogenicity, and efficacy of current BCG^4^.

Among said novel LAVs is BCGΔBCG1419c, for which we have developed and characterized two different versions, where the *BCG1419c* gene has been: (1) partially removed and replaced by a hygromycin resistance gene (first generation version^5^) or (2) completely deleted with no resistance marker incorporated (second generation version^6^). *BCG1419c* is predicted to encode for a c-di-GMP phosphodiesterase, therefore leading us to hypothesize that BCGΔBCG1419c might produce more c-di-GMP than its parental BCG, as we showed to occur in a growth-phase dependent-manner in planktonic cultures^7^. Of note, determination of c-di-GMP levels was reported by indirect measurement based on the activity of a reporter gene in said work.

We showed that the first-generation version of BCGΔBCG1419c increased in vitro biofilm production, while it lacked production of phthiocerol dimycocerosates (PDIM) and produced longer phenol glycolipid (PGL) species when cultured as biofilms in Sauton medium with no detergent^5^. Transcriptional profiling of this same version of BCGΔBCG1419c also in biofilm cultures showed decreased expression of genes involved in mycolic acids (MAs) metabolism and antigenic chaperones^8^ compared with BCG. These non-clinical characterizations were conducted with biofilm cultures because of overarching hypothesis is that in vitro biofilms produced by mycobacteria resemble yet not fully explored aspects of TB pathogenesis, which should be considered as an alternate approach to produce a vaccine candidate against this disease^9^. In fact, biofilm-like structures were recently observed in TB lesions from mice, guinea pigs and humans^10^, therefore strengthening the validity of our further development of BCGΔBCG1419c.

Based on this background, here we evaluated colonial morphology, biofilm production and lipidomic profile of planktonic and biofilm cultures of the second-generation version of BCGΔBCG1419c and its parental BCG Pasteur ATCC 35734 strain. The lipidomic analyses included evaluation of PDIM production, as we previously found no transcriptional changes in in vitro produced biofilm cultures^8^, therefore complicating an explanation for the absence of this compound in the hygromycin-marked BCGΔBCG1419^5^.

On the other hand, considering that most preclinical assays of novel LAV are conducted with bacteria cultured in planktonic conditions, employing shaken cultures in Middlebrook 7H9 medium with ADC/OADC supplement and detergent, in this work we decided to characterize the transcriptional profile of BCGΔBCG1419c and BCG when cultured in conditions that our group has recently employed to characterize the genome^11^, safety, immunogenicity, efficacy, and proteome of the antibiotic-less, second-generation version of BCGΔBCG1419c^7^. We went further in this work to directly assess c-di-GMP production in vitro. Finally, as we previously found transcriptional changes in the expression of genes involved in mycolic acids synthesis in biofilm cultures^8^, which can be incorporated into trehalose dimycolate (TDM, also known as cord factor) signaling via Mincle^12^ and Myd88^13^, we decided to compare the capacity of these strains to induce TNF-α, IL-6 and G-CSF in primary, bone-marrow derived murine macrophages obtained from wild type mice and knock-outs (KO) in Mincle or Myd88.

Our results confirm that some microbiological and transcriptomic differences already found to occur in the first-generation version of BCGΔBCG1419c compared with BCG are maintained in the antibiotic-less version of this vaccine candidate, except for changes in production of PDIM. This work also added additional information about transcriptional adaptation in BCG to the absence of *BCG1419c*, further suggesting some of these changes occur regardless of the cells being within mature biofilms or as planktonic cultures whereas other changes are likely dependent on the growth conditions the bacterial cells are encountered. Finally, we observed that for full induction of TNF-α, IL-6 or G-CSF by bone-marrow derived macrophages, both BCGΔBCG1419c and BCG required Mincle and Myd88.

## Results

### Microbiological characterization and increased c-di-GMP content in planktonic cultures compared with BCG of the antibiotic-less version of BCG∆BCG1419c

**Figure 1:**
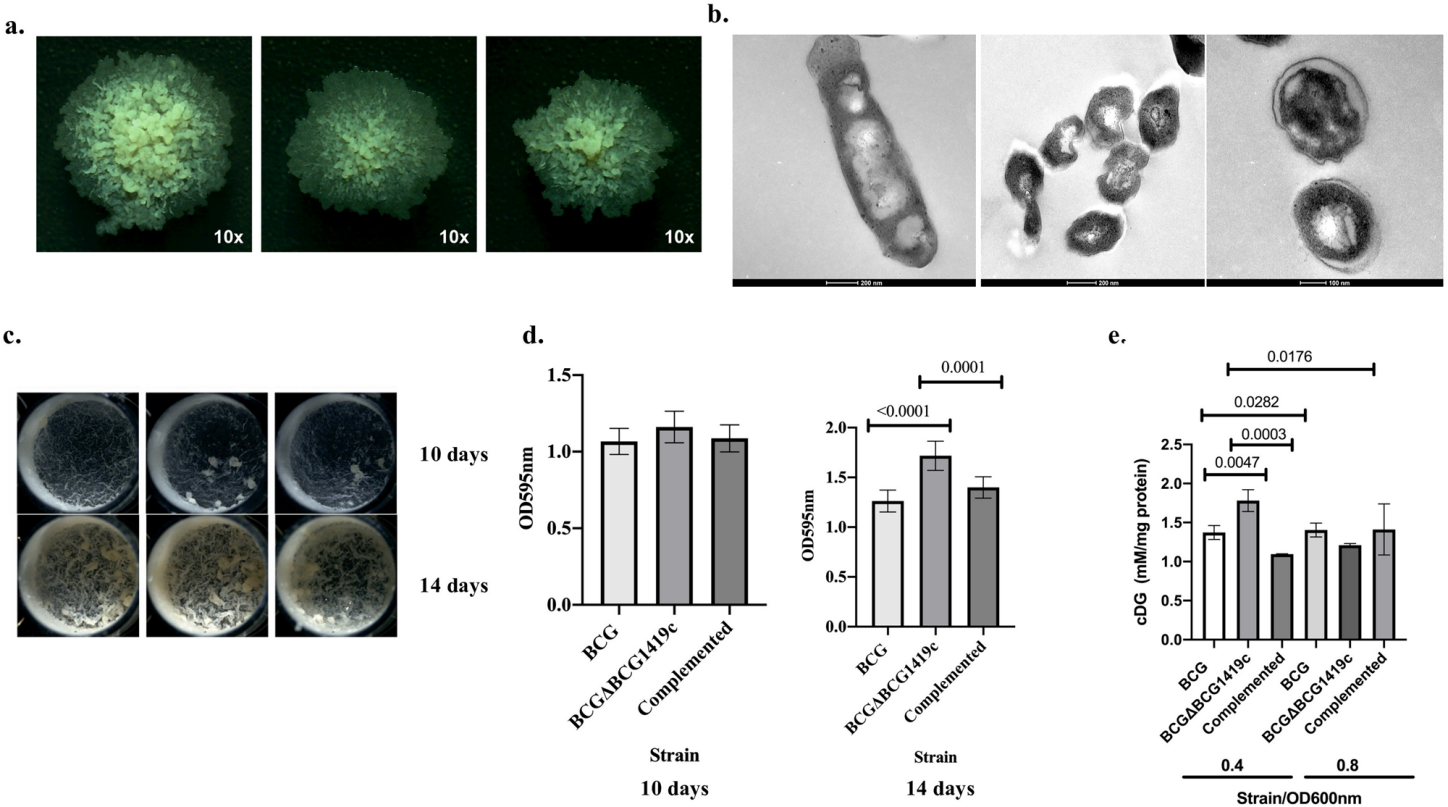
Phenotypic changes in colonial and ultrastructural morphology, biofilm and c-di-GMP production in the presence or absence of *BCG1419c*. (**a**) Isolated, single colonies obtained after 3 weeks of incubation at 37 °C on 7H10 OADC agar plates. (**b**) TEM of BCG and BCGΔBCG1419c planktonic cells cultured in 7H9 OADC Tween 80 and harvested at OD600nm 0.8. Representative electron microscopy micrographs of parental BCG and mutant BCGΔBCG1419c. (Left panel) typical morphology of the BCG strain. (Middle panel) abnormal morphology of BCGΔBCG1419c showing smaller bacilli with irregular shape, some cells show constrictions or cavities on the bacterial surface (arrows). (Right panel) high power magnification of mutant bacilli showing widened electron-lucid cell wall (arrows). (**c**) Surface pellicles formed in Sauton media with no detergent at 37 °C, 5% CO_2_, for 10 (top panel) or 14 days (bottom panel) in tissue culture flasks with vented caps. (**d**) Biofilm quantification of the different BCG strains at 10 days or 14 days of culture in Sauton media, in 48-well plates. For colonies, images at ×10 are shown. All experiments were performed three different times, with duplicates (**c**,**d**), and one representative image is shown in all instances; error bars represent standard deviations of the. One-Way ANOVA followed by Dunnett’s multiple comparison test was used to assess significance of changes among BCG strains. Statistically significant p actual values are shown on top of the bars depicting the means. (**e**) c-di-GMP content (mM) was normalized to mg of protein per sample and was determined by HPLC for in vitro cultures of BCG, BCGΔBCG1419c, and its complemented strain, at OD600 nm 0.4 and 0.8 (triplicate cultures). Data are shown as means with bars indicating standard deviation (SD). OD600nm refers to the optical density at 600 nm at which samples were harvested. Statistically significant differences are indicated by the p values shown.

As we have mentioned before, we constructed a novel, antibiotic-less version of the BCGΔBCG1419c vaccine candidate based on the fact that attenuation without the presence of antibiotic-resistance markers is required to fulfill the Geneva consensus criteria for novel TB vaccine candidates^14^. In an attempt to determine which phenotypes already reported for the hygromycin-marked version of BCGΔBCG1419c are maintained in the antibiotic-less version, we decided to evaluate colony morphology and biofilm production in vitro. We observed that the BCG Pasteur ATCC 35734 strain showed a greater elevation starting from the center and decreasing its density when reaching the edge. The edges of this strain were irregular and the shape of the colonies of this strain were rounder compared to the mutant and complemented strain (Fig. 1a, left panel) As for the BCG∆BCG1419c strain, colonies were flatter and thinner, their highest elevation was in the center with a wide edge, which was also irregular. The shape of this colony tended to be ovoid (Fig. 1a, middle panel). As for the complemented strain, it was smaller in size than the previous two strains, its center presented greater elevation that was not maintained most of the colony as opposed to BCG Pasteur WT strain, therefore restoring partially colonial morphology (Fig. 1a, right panel). An additional characterization we conducted this time was evaluating bacterial ultrastructure by Transmission Electron Microscopy (TEM). Here, we found that the morphology of BCGΔBCG1419c showed anomalies in comparison with the parent BCG Pasteur strain. BCGΔBCG1419c showed a smaller size and irregular shape with constriction and concavities of the cell wall, which was widened and electron-lucid in some bacteria, while in others the cell wall was thinner than that of the parent BCG strain (Fig. 1b).

We also compared the amount of biofilm produced at days 10 and 14 of incubation. Even though no major morphological differences were found (Fig. 1c), crystal violet staining allowed us to confirm that BCG∆BCG1419c produced more biofilm than parental BCG but only at day 14 post-incubation (Fig. 1d, p < 0.0001 for BCG∆BCG1419c vs BCG, and 0.0001 for BCG∆BCG1419c vs complemented strain, respectively).

On the other hand, because of the deletion of *BCG1419c*, we expected BCG∆BCG1419c to modify its content of the second messenger c-di-GMP. We indirectly confirmed this by means of the activity of a reporter gene^7^. To directly evaluate whether this nucleotide was affected by the presence or absence of *BCG1419c*, we quantitated this molecule by HPLC. As can be seen in Fig. 1e, we found that, in comparison to parental BCG, BCG∆BCG1419c had an increased amount of c-di-GMP at OD600nm 0.4 (p = 0.0047), an increase that was also observed when compared to its complemented derivative (p = 0.0003), suggesting a reversion to a wild-type like phenotype. However, this was partial as BCG had higher c-di-GMP amount compared with the complemented strain (p = 0.0282). Of note, the increased amount of c-di-GMP observed at OD600nm 0.4 in BCG∆BCG1419c showed a significant decrease when this strain reached OD600nm 0.8 (p = 0.0176), therefore showing that BCG∆BCG1419c indeed produced more c-di-GMP than BCG but only early in its growth in planktonic conditions.

### BCG∆BCG1419c does not show alterations in cell envelope lipid components compared with BCG Pasteur ATCC 35734

**Figure 2:**
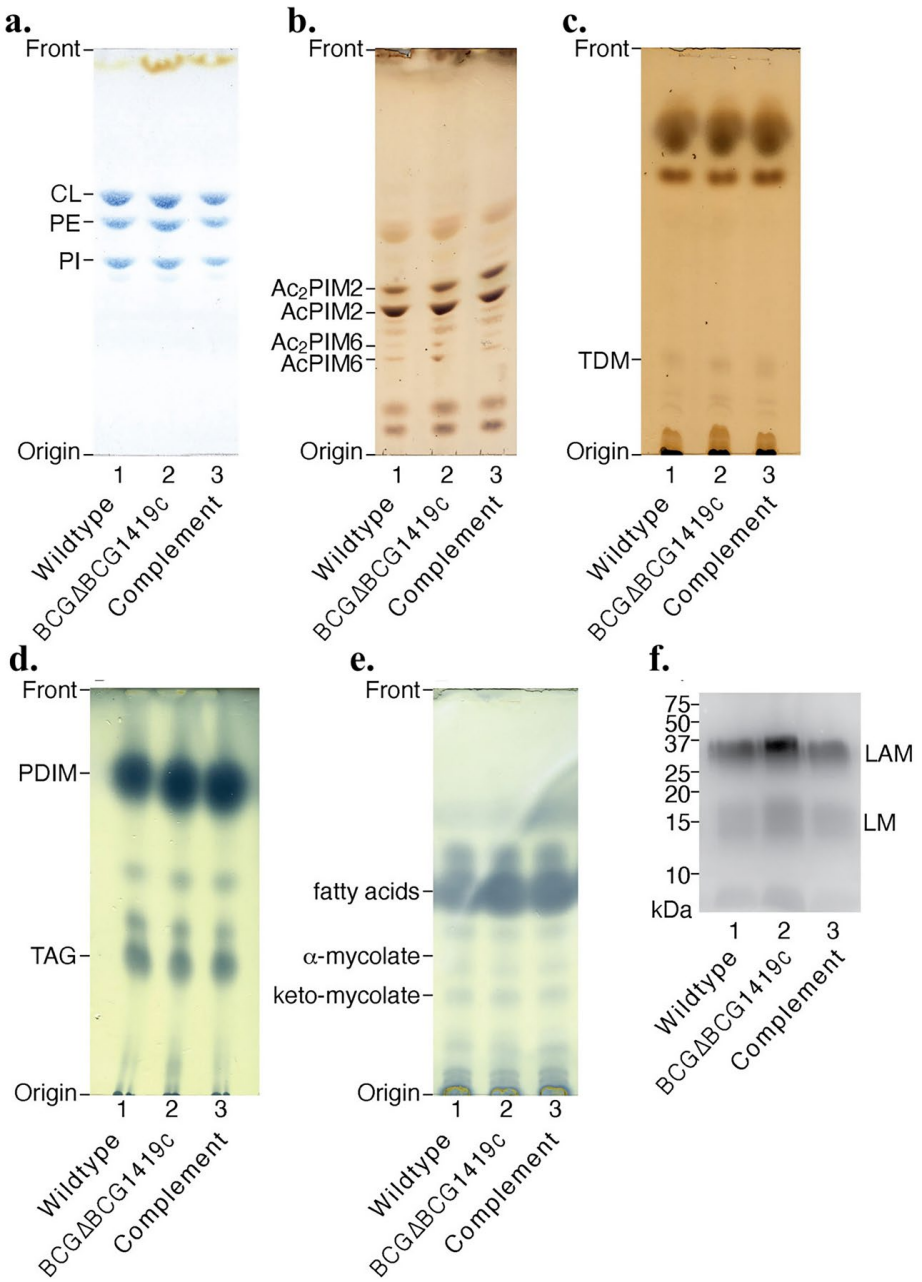
Analysis of cell envelope lipids. The lipids of the different BCG strains sampled from planktonic cultures in 7H9 OADC 0.05% Tween 80 at OD600nm 0.8 were extracted and analyzed by HPTLC as described in “Methods”. (**a**) Phospholipids. (**b**) PIMs. (**c**) TDM. (**d**) PDIMs and TAGs. (**e**) FAMEs and MAMEs. (**f**) LM/LAM. Analyses was performed with triplicate samples and a representative image is shown for each lipid class.

The first generation, hygromycin-resistant version of BCGΔBCG1419c lacked PDIM production in biofilm cultures^5^, although we did not find transcriptional changes between that mutant and its parental BCG strain that could explain the lack of this compound^8^. The mycobacterial plasma membrane is composed of major glycerophospholipids such as cardiolipin (CL), phosphatidylethanolamine (PE), and phosphatidylinositol (PI), therefore we characterized the production of these lipids in the second-generation BCGΔBCG1419c and its parental BCG Pasteur ATCC 35734 strain. For this, we extracted cellular lipids and analyzed them by high-performance thin layer chromatography. There were no obvious differences in the major plasma membrane phospholipids between the wildtype and BCG∆BCG1419c (Fig. 2a). Major glycolipid species such as AcPIM2, Ac_2_PIM2, AcPIM6, and A_c_2PIM6 were also detected at comparable levels between the wildtype and the mutant (Fig. 2b). The outer membrane of mycobacteria, known as mycomembrane, is composed of trehalose dimycolates (TDM) (Fig. 2c), phthiocerol dimycocerosates (PDIM), and triacylglycerols (TAG) (Fig. 2d) among other lipids. These outer membrane lipids were also detected in the mutant at comparable levels to the wildtype. Total contents of fatty acids and mycolic acids were analyzed as methyl ester derivatives (α- and keto-mycolic acids) and were also not significantly different between the wildtype and the mutant (Fig. 2e). Mannose-based lipoglycans such as lipomannan (LM) and lipoarabinomannan (LAM) are uniquely found in *Mycobacterium* species. We observed a slightly increased amount of LAM and LM in BCGΔBCG1419c compared with the wild type and complemented strains, although we did not determine whether this was quantitatively significant or not (Fig. 2f). While we cannot exclude the possibility that subtle structural features of cell envelope lipids are altered in the mutant, our analysis did not detect any differences in major cell envelope lipids in BCG∆BCG1419c compared with BCG Pasteur ATCC 35734 in biofilm cultures.

We next compared the production of these lipids when BCG∆BCG1419c and BCG Pasteur ATCC 35734 were cultured as planktonic cells in 7H9 OADC Tween 80 or mature (2 weeks-old) biofilm cultures in Sauton media with no detergent. Overall, we did not detect changes in cardiolipin (CL), phosphatidylethanolamine (PE), phosphatidylinositol (PI, supplementary Fig. 1a), Ac_2_PIM2, and AcPIM2, while A_c_2PIM6 and AcPIM6 seemed to differ in their content in planktonic versus biofilm cells but in a strain-independent manner (supplementary Fig. 1b). A slight variation could be observed in TDM present in BCG∆BCG1419c compared to parental BCG in both planktonic and biofilm cultures (supplementary Fig. 1c) although we cannot claim this to be significant as more precise analyses would need to be conducted. All the original TLC plates comparing planktonic versus biofilm cells (lipids) and SDS-PAGE (15% gel) visualized by Pro-Q Emerald 488 Glycoprotein Gel and Blot Stain Kit (Thermo Fisher) (for LM/LAM) are shown in Supplementary Fig. 1.

### Transcriptional profiling of BCG∆BCG1419c and BCG during planktonic growth conditions

We previously reported the transcriptional profile differences occurring between the first generation, hygromycin resistant version of BCG∆BCG1419c and its parental strain, BCG Pasteur 1173P2 when grown as biofilms in vitro^8^. Considering that we produced a second-generation, antibiotic-less version of BCG∆BCG1419c, now in BCG Pasteur ATCC 35734, for further vaccine development^15^, coupled with the fact that most preclinical studies of mycobacteria-based vaccine safety and/or efficacy are conducted with bacteria grown in planktonic conditions using Middlebrook 7H9 media with 10% ADC/OADC and 0.05% Tween 80, here we decided to investigate the transcriptional response of antibiotic-less BCG∆BCG1419c and its parental strain BCG Pasteur ATCC 35734 grown in planktonic conditions and harvesting cells for RNA isolation at the same stage (OD600nm 0.8) recently reported in an efficacy study against *M. tuberculosis* HN878^7^.

The BCG Pasteur 1173P2 genome, used as a reference, has 4109 protein and RNA-encoding genes. Here, we were able to detect gene transcription from all BCG genes (Supplementary Table 1). The most significant functional clusters were defined by DAVID^16^ indicating significant enrichments for three different functions, being downregulation of transmembrane (72 genes), arginine biosynthesis (7 genes), and cell wall organization (12 genes, Benjamini–Hochberg adjusted p values 0.00026, 0.0015, and 0.036, respectively) in BCG∆BCG1419c compared with its parental strain BCG Pasteur ATCC 35734 (Supplementary Table 1).

Regarding differential gene expression [considered as significant (when both Log_2_-fold change ≥ 0.585 or ≤  − 0.585 plus p < 0.05) or not] overall, we found relatively few differences and mostly gene downregulation (36 gene upregulated, 123 genes downregulated, Table 1). Of note, among the 123 downregulated genes, we observed that 13 genes belong to the MtrA regulon, and 4 genes belongs to the ArgR regulon (Table 1). Among the genes that were significantly upregulated in BCGΔBCG1419c compared with BCG WT (Log_2_ Fold-change ≥ 0.585 plus p < 0.05) we found *rrs* (16S rRNA), *rpsN1* (30S ribosomal protein), *rrl* (23S rRNA), 5S rRNA, *rpmC* (50S ribosomal protein), *glyU* (tRNA Gly), *serT* (tRNA Ser), *groES*, *mbtH*, *BCG_3249c* (a possible anti-sigma factor RshA regulates the activity of the mycobacterial stress response sigma factor SigH), *BCG_1418c* (hypothetical protein) and *BCG_1420* (LuxR family transcriptional regulator, with an adenylate/guanylate cyclase domain, an ATPase domain, and a helix-turn-helix DNA-binding domain) (Table 1).

**Table 1.** Differentially expressed genes with significant changes found between BCGΔBCG1419c and BCG in planktonic cultures.

Upregulated in BCGΔBCG1419c vs BCG WT fold-change ≥ 0.585 plus p < 0.05						
GENE_NAME	GENE_ID	Mtb ortholog	Product	LOG2FOLD	AVG_PVALUE	Regulon
rrs	rrs		16S rRNA	3.4982	0.000104738	
EBG00001157317	EBG00001157317		SSU_rRNA_archaea	2.9089	0.000387952	
EBG00001157343	EBG00001157343		PK-G12rRNA	2.2264	0.034778283	
secE2	BCG_0417	Rv0379	Possible protein transport protein secE2 with Calcium dodecin domain	1.6343	0.012587538	
EBG00001157360	EBG00001157360		5_8S_rRNA	1.5695	0.005169291	
BCG_3831c	BCG_3831c		Probable remnant of a transposase	1.4757	0.049151559	
BCG_3079c	BCG_3079c	Rv3054c	Conserved hypothetical protein with NAD(P)H-dependent FMN reductase domain	1.3741	0.000116064	
EBG00001157348	EBG00001157348		LSU_rRNA_bacteria	1.2775	0.005406273	
rpsN1	BCG_0767	Rv0717	Probable 30S ribosomal protein S14 rpsN1	1.2582	0.015651912	
BCG_2010c	BCG_2010c	Rv1993c	Conserved hypothetical protein	1.1703	0.013705724	
rrl	rrl		23S rRNA	1.1078	0.016674466	
groES	BCG_3488c	Rv3418c	10 kDa chaperonin groES	1.098	0.020911838	
EBG00001157315	EBG00001157315		5S_rRNA	1.05	0.031607557	
BCG_2922c	BCG_2922c	Rv2901c	Conserved hypothetical protein	1.0287	0.037531455	
mbtH	BCG_2391c	Rv2377c	Putative conserved protein mbtH	1.004	0.011346227	
BCG_0026c	BCG_0026c	Rv3920c	Hypothetical protein similar to jag protein	0.9649	0.02941768	
phoY1_1	BCG_3330c	**Rv3301c**	Probable phosphate-transport system transcriptional regulatory protein phoU homolog 1 phoY1	0.9622	0.007044978	
PE_PGRS43b	BCG_2509c	Rv2490c	PE-PGRS family protein [second part]	0.9184	0.047561816	
rpmC	BCG_0759	Rv0709	Probable 50S ribosomal protein L29 rpmC	0.9129	0.041923764	
glyU	glyU		tRNA-Gly	0.9079	0.000861898	
phoY1_2	BCG_3366c	**Rv3301**c	Probable phosphate-transport system transcriptional regulatory protein phoU homolog 1 phoY1	0.8864	0.010088291	
BCG_2220c	BCG_2220c	Rv2204c	Conserved hypothetical protein with iron-sulfur cluster assembly accessory protein domain	0.8801	0.04788665	
BCG_0840	BCG_0840	Rv0787A	Conserved hypothetical protein with phosphoribosylformylglycinamidine (FGAM) synthase domain	0.8727	0.029136225	
BCG_1418c	BCG_1418c	Rv1356c	Hypothetical protein	0.8671	0.000299023	
serT	serT		tRNA-Ser	0.8631	0.000637902	
BCG_2827	BCG_2827	Rv2809	Hypothetical protein	0.8464	0.041819341	
PE_PGRS22	BCG_1151	Rv1091	PE-PGRS family protein	0.8331	0.028777497	
PE_PGRS45b	BCG_2641c	Rv2615c	PE-PGRS family protein	0.7918	0.012061469	
TB7.3_1	BCG_3248c	**Rv3221c**	Biotinylated protein TB7.3 contains acetyl-CoA carboxylase biotin carboxyl carrier protein subunit domain	0.791	0.013039128	
TB7.3_2	BCG_3341c	**Rv3221c**	Biotinylated protein TB7.3 contains acetyl-CoA carboxylase biotin carboxyl carrier protein subunit domain	0.7188	0.019029613	
BCG_2053	BCG_2053	Rv2034	Probable ArsR-type repressor protein	0.6551	0.018687862	
BCG_1420	BCG_1420	Rv1358	Probable transcriptional regulatory protein	0.6283	0.000717219	
BCG_3348c	BCG_3348c	Rv3225c	Possible transferase, GCN5-related N-acetyltransferase, phosphorylase domain	0.6262	0.003293885	
BCG_3227c	BCG_3227c	Rv3202c	Possible ATP-dependent DNA helicase	0.5937	0.000595553	
BCG_3249c	BCG_3249c	Rv3221A	Possible anti-sigma factor RshA regulates the activity of the mycobacterial stress response sigma factor SigH	0.5886	0.042051519	
gid_1	BCG_3977c		Probable glucose-inhibited division protein B gid	0.5873	0.421759769	

Downregulated BCGΔBCG1419c vs BCG WT fold-change ≤ − 0.585 plus p < 0.05						
GENE_NAME	GENE_ID	Mtb ortholog	Product	LOG2FOLD	AVG_PVALUE	Regulon
BCG_3715c	BCG_3715c	Rv3657c	Possible conserved alanine rich membrane protein	− 1.1444	0.023526035	
BCG_3154	BCG_3154	Rv3131	Conserved hypothetical protein. Putative nitroreductase, member of the DosR-regulon	− 1.0879	0.000785114	
cobLa	BCG_2092c		Probable precorrin-6y methyltransferase CobLa [first part]	− 1.0652	0.047535781	
BCG_0295	BCG_0295	Rv0257	Conserved hypothetical protein	− 0.9771	0.027386067	
BCG_0788	BCG_0788	Rv0738	Conserved hypothetical protein with DNA damage-inducible protein DinB domain	− 0.9704	0.009118011	
BCG_1419c	BCG_1419c	Rv1357c	Conserved hypothetical protein with cyclic diguanylate phosphodiesterase domain	− 0.9519	0.009820681	
pirG	BCG_3872	Rv3810	Exported repetitive protein precursor pirG/erp	− 0.9464	0.000708503	MtrA regulon
lipF	BCG_3551c	Rv3487c	Probable esterase/lipase lipF	− 0.9198	0.000144981	
BCG_1496c	BCG_1496c	Rv1435c	Probable conserved Proline, Glycine, Valine-rich secreted protein	− 0.9068	0.006275716	MtrA regulon
BCG_1462	BCG_1462	Rv1401	Possible membrane protein	− 0.8878	0.002947653	
BCG_3695	BCG_3695		Putative transposase	− 0.8857	0.041641661	
murG	BCG_2170c	Rv2153c	UPD-N-acetylglucosamine-N-acetylmuramyl-(pentapeptide) pyrophosphoryl-undecaprenol-N-acetylglucosamine transferase MurG	− 0.8737	0.009443607	
BCG_2763	BCG_2763	Rv2747	Possible transferase, Probable L-glutamate alpha-N-acetyltranferase ArgA (alpha-N-acetylglutamate synthase)	− 0.8719	0.005494031	
BCG_0711c	BCG_0711c	Rv0662c	Conserved hypothetical protein Possible antitoxin VapB7	− 0.865	0.031849363	
lpqZ	BCG_1304	Rv1244	Putative lipoprotein lpqZ	− 0.8632	0.004945189	
serA2	BCG_0778c	Rv0728c	Possible D-3-phosphoglycerate dehydrogenase serA2	− 0.8559	0.011718358	
BCG_3153c	BCG_3153c	Rv3130c	Conserved hypothetical protein Triacylglycerol synthase (diacylglycerol acyltransferase) Tgs1	− 0.8421	0.034329997	
nirD	BCG_0291	Rv0253	Probable nitrite reductase [NAD(P)H] small subunit nirD	− 0.8362	0.018275726	
BCG_0518	BCG_0518	Rv0477	Possible conserved secreted protein	− 0.8313	0.005587559	
lipR	BCG_3109	Rv3084	Probable acetyl-hydrolase/esterase lipR	− 0.828	0.010400861	
argB	BCG_1693	Rv1654	Probable Acetylglutamate kinase argB	− 0.8205	0.003955123	ArgR regulon
BCG_0699	BCG_0699	Rv0650	Possible sugar kinase with ROK family protein domain	− 0.8087	0.027255813	
BCG_1762	BCG_1762	Rv1723	Probable hydrolase with CubicO group peptidase, beta-lactamase class C family domain	− 0.8087	0.009155488	
BCG_3465	BCG_3465	Rv3395A	Probable membrane protein	− 0.7883	0.035013964	
BCG_0008c	BCG_0008c	Rv0008c	Possible membrane protein with cell wall synthesis protein CwsA domain	− 0.7879	0.037588749	
BCG_1219c	BCG_1219c	Rv1158c	Conserved hypothetical ala-, pro-rich protein	− 0.7862	0.005061423	MtrA regulon
BCG_0702c	BCG_0702c	Rv0653c	Possible transcriptional regulatory protein (probably tetR-family)	− 0.7705	0.024929043	
BCG_0501	BCG_0501	Rv0461	Probable transmembrane protein	− 0.7693	0.005563857	
BCG_2782	BCG_2782	Rv2765	Probable alanine rich hydrolase with dienelactone hydrolase family protein domain	− 0.7689	0.026772117	
speE	BCG_2625	Rv2601	Putative spermidine synthase speE	− 0.7643	0.008314323	
BCG_0673	BCG_0673	Rv0627	Conserved hypothetical protein Possible toxin VapC5	− 0.7584	0.010403475	
BCG_3664c	BCG_3664c	Rv3600c	Conserved hypothetical protein with type III pantothenate kinase domain	− 0.7558	0.009655975	
yrbE1A	BCG_0204	Rv0167	Conserved hypothetical integral membrane protein yrbE1A	− 0.7506	0.004801835	
adhE1	BCG_0198c	Rv0162c	Putative zinc-type alcohol dehydrogenase (e subunit) adhE	− 0.7478	0.024960044	
BCG_1844	BCG_1844	Rv1810	Conserved hypothetical protein	− 0.7437	0.011342536	
purT	BCG_0426	Rv0389	Probable phosphoribosylglycinamide formyltransferase 2 purT	− 0.7374	0.002402391	
argF	BCG_1695	Rv1656	Probable Ornithine carbamoyltransferase, anabolic ArgF	− 0.7363	0.010148525	ArgR regulon
BCG_2741c	BCG_2741c	Rv2728c	Conserved hypothetical alanine rich protein with class III extradiol ring-cleavage dioxygenase family protein domain	− 0.7323	0.016000596	
BCG_0284	BCG_0284	Rv0246	Probable conserved integral membrane protein	− 0.72	0.027263273	
cobD	BCG_2253c	Rv2236c	Probable conserved membrane protein CobD	− 0.7136	0.033002992	
deoD	BCG_3372	Rv3307	Probable purine nucleoside phosphorylase deoD	− 0.7087	0.006876059	
pks16	BCG_1070	Rv1013	Putative polyketide synthase pks16	− 0.7077	0.003716129	
BCG_1059c	BCG_1059c	Rv1002c	Conserved membrane protein with dolichyl-phosphate-mannose–protein mannosyltransferase domain	− 0.707	0.005819792	
xerC	BCG_2915c	Rv2894c	Probable integrase/recombinase xerC	− 0.7054	0.009092191	MtrA regulon
BCG_0891	BCG_0891	Rv0839	Conserved hypothetical protein with class I SAM-dependent methyltransferase domain	− 0.7016	0.001141476	
BCG_1349c	BCG_1349c	Rv1290c	Conserved hypothetical protein	− 0.7016	0.005788882	
celA1	BCG_0093	Rv0062	Possible cellulase celA1 (ENDOGLUCANASE)	− 0.7013	0.011925226	
BCG_1518c	BCG_1518c	Rv1457c	Probable unidentified antibiotic-transport integral membrane ABC transporter	− 0.7011	0.017966363	
BCG_0352	BCG_0352	Rv0312	Conserved hypothetical proline and threonine rich protein with Hsp70 family protein domain	− 0.7007	0.006180045	MtrA regulon
BCG_0517	BCG_0517	Rv0476	Possible conserved transmembrane protein	− 0.7003	0.008443131	
argJ	BCG_1692	Rv1653	Probable Glutamate n-acetyltransferase argJ	− 0.6962	0.011937422	ArgR regulon
BCG_1061c	BCG_1061c	Rv1004c	Probable membrane protein	− 0.6961	0.028415351	
prrA	BCG_0955c	Rv0903c	Two component response transcriptional regulatory protein prrA	− 0.6901	0.002751943	
BCG_0934	BCG_0934	Rv0882	Probable transmembrane protein	− 0.6794	0.038109383	
BCG_2713	BCG_2713	Rv2700	Possible secreted alanine rich protein with LytR cell envelope-related transcriptional attenuator domain	− 0.6777	0.015900774	
BCG_1310	BCG_1310	Rv1250	Probable drug-transport integral membrane protein	− 0.6758	0.013796646	
BCG_3054	BCG_3054	Rv3031	Conserved hypothetical protein with glycoside hydrolase family 57 protein domain	− 0.6735	0.012038058	
add	BCG_3379c	Rv3313c	Probable adenosine deaminase add	− 0.6725	0.022782705	
panC	BCG_3666c	Rv3602c	Probable pantoate–beta-alanine ligase panC	− 0.6717	0.008371359	
BCG_1539	BCG_1539	Rv1477	Hypothetical invasion protein (ripA)	− 0.6698	0.00493817	MtrA regulon
ephC	BCG_1185	Rv1124	Probable epoxide hydrolase ephC	− 0.6668	0.024392971	
BCG_0265	BCG_0265	Rv0228	Probable integral membrane acyltransferase	− 0.6665	0.031158919	
BCG_1619c	BCG_1619c	Rv1566c	Possible inv protein	− 0.6661	0.007192759	
BCG_1001c	BCG_1001c		Probable mycolyl transferase	− 0.6654	0.045990406	
ugpBa	BCG_2853c	Rv2833c	Probable Sn-glycerol-3-phosphate-binding lipoprotein ugpB [first part]	− 0.6645	0.035959294	
BCG_1624	BCG_1624	Rv1571	Conserved hypothetical protein with 2′-5′RNA ligase family protein domain	− 0.663	0.035162251	
sugI	BCG_3401	Rv3331	Probable sugar-transport integral membrane protein sugI	− 0.6629	0.018548577	
BCG_3854	BCG_3854	Rv3792	Probable conserved transmembrane protein Arabinofuranosyltransferase AftA	− 0.6629	0.007871683	
BCG_3676c	BCG_3676c	Rv3612c	Conserved hypothetical protein	− 0.6601	0.003454172	
dedA	BCG_2664	Rv2637	Possible transmembrane protein dedA	− 0.6587	0.039418929	
BCG_3491c	BCG_3491c	Rv3421c	Conserved hypothetical protein with tRNA (adenosine(37)-N6)-threonylcarbamoyltransferase complex dimerization subunit type 1 TsaB domain	− 0.6525	0.031652896	
BCG_1863	BCG_1863	Rv1828	Conserved hypothetical protein with MerR family transcriptional regulator domain	− 0.6512	0.007243893	
BCG_0842c	BCG_0842c	Rv0789c	Hypothetical protein	− 0.65	0.042091095	
PPE9	BCG_0425c		PPE family protein	− 0.6477	0.024967853	
cut4	BCG_3518	Rv3452	Probable cutinase precursor cut4	− 0.6474	0.02589246	
BCG_1218c	BCG_1218c	Rv1157c	Conserved hypothetical ala-, pro-rich protein	− 0.6467	0.006357262	MtrA regulon
BCG_3691	BCG_3691	Rv3633	Conserved hypothetical protein with Ectoine hydroxylase-related dioxygenase, phytanoyl-CoA dioxygenase domain	− 0.6456	0.004480936	
cobS	BCG_2224	Rv2208	Probable cobalamin 5'-phosphate synthase CobS	− 0.6448	0.020180231	
BCG_3851	BCG_3851	Rv3789	Conserved hypothetical protein with GtrA domain-containing protein	− 0.6412	0.02717627	
echA16	BCG_2851	Rv2831	Probable enoyl-CoA hydratase echa16	− 0.6407	0.040951984	
BCG_3745c	BCG_3745c	Rv3686c	Conserved hypothetical protein	− 0.6383	0.023684945	
BCG_0241c	BCG_0241c	Rv0204c	Probable conserved transmembrane protein	− 0.6362	0.010034117	
BCG_3015	BCG_3015	Rv2994	Probable conserved integral membrane protein with MFS transporter domain	− 0.6361	0.016319392	
leuB	BCG_3016c	Rv2995c	3-isopropylmalate dehydrogenase leuB	− 0.636	0.02535765	
BCG_1657	BCG_1657	Rv1619	Conserved membrane protein	− 0.6359	0.029269187	
argD	BCG_1694	Rv1655	Probable Acetylornithine aminotransferase argD	− 0.635	0.010948399	ArgR regulon
fadD33	BCG_1407	Rv1345	Possible polyketide synthase fadD33	− 0.6338	0.007716787	
BCG_1724c	BCG_1724c	Rv1686c	Probable conserved integral membrane protein ABC transporter	− 0.6334	0.03708873	
BCG_2589	BCG_2589	Rv2566	Long conserved hypothetical protein [second part]	− 0.6334	0.033570675	
BCG_0388	BCG_0388	Rv0349	Hypothetical protein	− 0.6315	0.025410477	
BCG_1540	BCG_1540	Rv1478	Hypothetical invasion protein (ripB)	− 0.6313	0.004572077	MtrA regulon
BCG_0330	BCG_0330	Rv0290	Probable conserved transmembrane protein 3 ESX conserved component EccD3. ESX-3 type VII secretion system protein	− 0.6302	0.015606343	
BCG_1711c	BCG_1711c	Rv1673c	Conserved hypothetical protein with Transglutaminase-like enzyme, putative cysteine protease domain	− 0.6289	0.043524332	
yrbE1B	BCG_0205	Rv0168	Conserved hypothetical integral membrane protein yrbE1B	− 0.6288	0.016757565	
narL	BCG_0896c	Rv0844c	Possible nitrate/nitrite response transcriptional regulatory protein narL	− 0.627	0.010270002	
BCG_0055	BCG_0055		Putative secreted protein P60-related protein [second part]	− 0.6268	0.026680451	
BCG_2812c	BCG_2812c	Rv2794c	Conserved hypothetical protein phosphopantetheinyl transferase PptT	− 0.6226	0.015274191	
cydD	BCG_1659c	Rv1621c	Probable 'component linked with the assembly of cytochrome' transport transmembrane ATP-binding protein ABC transporter cydD	− 0.6197	0.026880206	
BCG_3754	BCG_3754	Rv3695	Possible conserved membrane protein RDD family protein	− 0.6188	0.020087176	
BCG_2071c	BCG_2071c	Rv2052c	Conserved hypothetical protein	− 0.6154	0.018909791	
BCG_2196	BCG_2196	Rv2181	Probable conserved integral membrane protein Alpha(1→2)mannosyltransferase/manT	− 0.6154	0.007679435	
BCG_2692	BCG_2692	Rv2679	Probable enoyl-CoA hydratase echA15	− 0.6145	0.025973179	
pra	BCG_1136	Rv1078	Probable Proline-rich antigen homolog pra	− 0.6119	0.014551011	
mmsB	BCG_0802c	Rv0751c	Probable 3-hydroxyisobutyrate dehydrogenase mmsB	− 0.6115	0.008050415	
mtc28	BCG_0071c	Rv0040c	Secreted proline rich protein MTC28 (PROLINE RICH 28 KDA ANTIGEN)	− 0.6097	0.005413412	MtrA regulon
BCG_3443	BCG_3443	Rv3371	Conserved hypothetical protein possible triacylglycerol synthase (diacylglycerol acyltransferase)	− 0.6089	0.006884392	
arsB2	BCG_3643	Rv3578	Possible arsenical pump integral membrane protein arsB2	− 0.6069	0.029200206	
mutA	BCG_1555	Rv1492	Probable methylmalonyl-CoA mutase small subunit mutA	− 0.6052	0.012106302	
menE	BCG_0586c	Rv0542c	Possible o-succinylbenzoic acid–CoA ligase menE	− 0.6049	0.01997206	
BCG_3879	BCG_3879	Rv3817	Putative phosphotransferase	− 0.6039	0.027736949	
BCG_1587	BCG_1587	Rv1535	Hypothetical protein	− 0.6026	0.024513436	
BCG_1802c	BCG_1802c	Rv1761c	Hypothetical exported protein	− 0.5977	0.028422702	
BCG_2479	BCG_2479	Rv2459	Probable conserved integral membrane transport protein MFS transporter	− 0.5973	0.007794219	
BCG_3777	BCG_3777	Rv3717	Conserved hypothetical protein with N-acetylmuramoyl-L-alanine amidase domain	− 0.5972	0.016730339	MtrA regulon
BCG_1618c	BCG_1618c	Rv1565c	Conserved hypothetical membrane protein with acyltransferase and SGNH domains	− 0.5945	0.013232892	MtrA regulon
BCG_0263c	BCG_0263c	Rv0226c	Probable conserved transmembrane protein	− 0.5931	0.009766941	
plcD	BCG_1794c	Rv2351c	Probable phospholipase C 4 plcD	− 0.5912	0.038388997	MtrA regulon
rfe	BCG_1362	Rv1302	Putative undecapaprenyl-phosphate alpha-n-acetylglucosaminyltransferase rfe/wecA	− 0.5904	0.032022205	
BCG_0697	BCG_0697	Rv0648	Alpha-mannosidase	− 0.588	0.007643403	
BCG_1575	BCG_1575	Rv1523	Probable methyltransferase	− 0.5879	0.027733217	
BCG_3514	BCG_3514	Rv3448	Probable conserved integral membrane protein	− 0.5864	0.044909115	
BCG_3772	BCG_3772	Rv3712	Possible ligase	− 0.5864	0.019375521	
sapM	BCG_3375	Rv3310	Possible acid phosphatase	− 0.5853	0.010551491	MtrA regulon

Genes highlighted in bold correspond to those matching potential pseudogenes/interrupted genes.

Genes that were significantly downregulated in BCGΔBCG1419c compared with BCG WT (Log_2_ Fold-change ≤  − 0.585 plus p < 0.05) included diverse functions, among others: *BCG_3154* (*Rv3131*, putative nitroreductase, member of the DosR-regulon), *BCG_1419c* (conserved hypothetical protein with cyclic diguanylate phosphodiesterase domain), *lipF* (Probable esterase/lipase), *murG* (UPD-N-acetylglucosamine-N-acetylmuramyl-(pentapeptide) pyrophosphoryl-undecaprenol-N-acetylglucosamine transferase), *argA* (alpha-N-acetylglutamate synthase), *serA2* (d-3-phosphoglycerate dehydrogenase), and *BCG_3153c* (*Rv3130c*, *tgs1*, triacylglycerol synthase, member of the DosR-regulon) (Table 1).

There was also downregulation of genes belonging to the MtrA regulon: *pirG* (exported repetitive protein precursor), *BCG_1496c* (probable conserved Proline, Glycine, Valine-rich secreted protein), *BCG_1219c* (conserved hypothetical ala-, pro-rich protein), *xerC* (probable integrase/recombinase), *BCG_0352* (conserved hypothetical proline and threonine rich protein with Hsp70 family protein domain), *BCG_1539* (hypothetical invasion protein, *ripA*), *BCG_1218c* (conserved hypothetical ala-, pro-rich protein), *BCG_1540* (hypothetical invasion protein, *ripB*), *mtc28* (secreted proline rich protein, proline rich 28 KDa antigen), *BCG_3777* (conserved hypothetical protein with N-acetylmuramoyl-l-alanine amidase domain), *BCG_1618c* (conserved hypothetical membrane protein with acyltransferase and SGNH domains), *plcD* (probable phospholipase C 4), and *sapM* (possible acid phosphatase) (Table 1).

Finally, we also observed downregulation of genes belonging to the ArgR regulon: *argB* (probable acetylglutamate kinase), *argF* (probable ornithine carbamoyltransferase), argJ (probable glutamate N-acetyltransferase), *argD* (probable acetylornithine aminotransferase) (Table 1).

### BCG∆BCG1419c does not show changes in macrophage activation via Mincle nor Myd88 compared with BCG as both mycobacteria require these molecules for full induction of TNF-α, IL-6 and G-CSF

**Figure 3:**
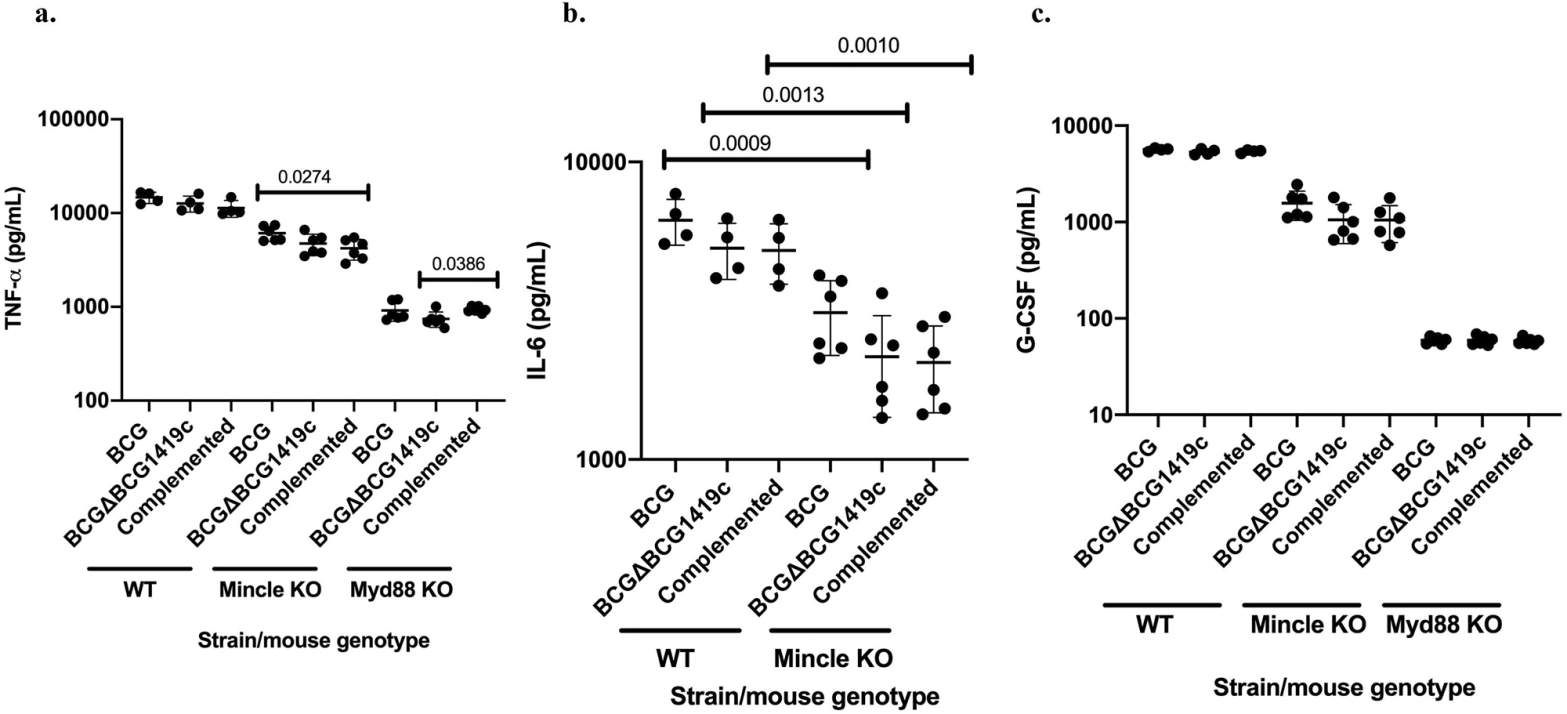
Cytokine secretion by wild type, Mincle- and Myd88-KO murine macrophages in response to infection with BCG, BCGΔBCG1419c and its complemented strain. BCG strains were obtained from planktonic cultures in 7H9 OADC 0.05% Tween 80 at OD600nm 0.8 and used to infect primary bone-marrow derived macrophages from mice as detailed in “Methods”. (**a**) TNF-α. (**b**) IL-6. (**c**) G-CSF. Macrophages were used for stimulation in duplicate or triplicate wells, giving an average cytokine values from 4 to 6 wells in each experiment. Bars indicate the mean cytokine values with individual values indicated as dots; error bars represent standard deviations of the mean. One-Way ANOVA followed by Dunnett’s multiple comparison test or Kruskal Wallis followed by Dunn’s multiple comparison test was used to assess significance of changes among BCG strains, depending on data distribution. An unpaired t test, Welch’s t test, or Mann–Whitney test was used to compare each cytokine produced by WT and each KO macrophage, depending on data distribution. Statistically significant p actual values are shown on top of the bars depicting the means.

Mycolic acids can be incorporated into trehalose dimycolate (TDM, cording factor), a highly immunostimulatory mycobacterial glycolipid that can signal via Mincle^12^ and Myd88^13^. As we previously found transcriptional changes in the expression of genes involved in mycolic acids synthesis in biofilm cultures of the first-generation version of BCGΔBCG1419c^8^, and even though we did not observe any significant change in the amount of TDM produced between BCG and BCGΔBCG1419c (Fig. 2c), we decided to compare the capacity of BCG, BCGΔBCG1419c and its complemented strain, to induce TNF-α, IL-6 and G-CSF in primary, bone-marrow derived murine macrophages obtained from wild type mice and knock-outs (KO) in Mincle or Myd88, because we cannot rule out that subtle structural features of TDM (i.e. cyclopropanation) could be altered in the mutant. We observed that production of TNF-α, IL-6, and G-CSF was reduced in KO- compared with WT macrophages, regardless of what strain was used to infect them (Fig. 3 and Table 2). The effect of lacking Mincle was statistically significant (two–fivefold decrease) in reducing secretion of TNF-α, IL-6, and G-CSF (Fig. 3 and Table 2; table shows comparison between WT and KO macrophages to avoid crowding Fig. 3a and c). The lack of Myd88 led to significant and even more pronounced decrease in secretion of cytokines (four to sixfold decrease for TNF-α and almost or above 90-fold for G-CSF, not detectable levels for IL-6) compared with WT macrophages (Fig. 3 and Table 2; table shows comparison between WT and KO macrophages to avoid crowding Fig. 3a and c). Together, these results show that macrophage activation via Mincle or Myd88 does not differ between BCG and BCGΔBCG149c as both strains required these receptors to fully activate macrophages to produce the proinflammatory cytokines determined here.

**Table 2.** Δ. Comparison of cytokines secretion by wild type, Mincle- or Myd88 KO primary macrophages in response to infection with BCG or BCGΔBCG1419c.

TNF-α (pg/mL)								
WT			Mincle KO			Myd88 KO		
BCG	BCGΔBCG1419c	Complemented	BCG	BCGΔBCG1419c	Complemented	BCG	BCGΔBCG1419c	Complemented
14,685.25 ± 2022.29	12,694.92 ± 2463.62	11,330.98 ± 2321.5	6101.66 ± 1108.66	4737.03 ± 1213.91	4206.09 ± 1057.24	917.235 ± 216.03	745.483 ± 138.99	937.91 ± 69.9
			p < 0.0001 (WT vs Mincle KO)	p = 0.0001 (WT vs Mincle KO)	p = 0.0095 (WT vs Mincle KO)	p = 0.0095 (WT vs Myd88 KO)	p = 0.0023 (WT vs Myd88 KO)	p = 0.0095 (WT vs Myd88 KO)
Values shown are the means with standard deviations						p = 0.0022 (Mincle KO vs Myd88KO)	p = 0.0004 (Mincle KO vs Myd88KO)	p = 0.00046 (Mincle KO vs Myd88KO)
Mean fold-change vs WT			2.4	2.7	2.7	6.6	6.3	4.5

IL-6 (pg/mL)								
WT			Mincle KO			Myd88 KO		
BCG	BCGΔBCG1419c	Complemented	BCG	BCGΔBCG1419c	Complemented	BCG	BCGΔBCG1419c	Complemented
6370 ± 1122.04	5128 ± 1099.71	5050.15 ± 1158.33	3114.88 ± 878.28	2214.77 ± 828.76	2120.979 ± 687.01	Not determined (ND, below detection limits)	ND	ND
			p < 0.0001 (WT vs Mincle KO)	p = 0.0001 (WT vs Mincle KO)	p = 0.0095 (WT vs Mincle KO)	ND	ND	ND
Values shown are the means with standard deviations								
Mean fold-change vs WT			2	2.3	2.4			

G-CSF (pg/mL)								
WT			Mincle KO			Myd88 KO		
BCG	BCGΔBCG1419c	Complemented	BCG	BCGΔBCG1419c	Complemented	BCG	BCGΔBCG1419c	Complemented
5639.35 ± 206.47	5339.43 ± 349.16	5412.86 ± 184.42	1570.77 ± 525.94	1055.72 ± 458.25	1048.38 ± 434.76	59.35 ± 4.53	59.38 ± 6.26	58.39 ± 4.53
			p < 0.0001 (WT vs Mincle KO)	p < 0.0001 (WT vs Mincle KO)	p < 0.0001 (WT vs Mincle KO)	p < 0.0001 (WT vs Myd88 KO)	p < 0.0001 (WT vs Myd88 KO)	p < 0.0001 (WT vs Myd88 KO)
Values shown are the means with standard deviations						p = 0.0009 (Mincle KO vs Myd88 KO)	p = 0.0031 (Mincle KO vs Myd88 KO)	p = 0.0026 (Mincle KO vs Myd88 KO)
Mean fold-change vs WT			3.6	5.1	5.2	95	89.9	92.7

## Discussion

*Mycobacterium bovis* Bacille Calmette-Guèrin (BCG) remains, after over one century now, as the only vaccine in use to fight the global burden imposed by TB. Given that BCG has been unable to reduce pulmonary TB, which accounts for roughly 80% cases of this disease, a number of vaccine candidates intended to replace or improve BCG have been developed, including live, attenuated, mycobacteria-based vaccine (LAV) candidates, which aim to increase safety, immunogenicity, and efficacy of current BCG^4^.

One of these LAV candidates is BCGΔBCG1419c, developed following the overarching hypothesis that in vitro biofilms produced by mycobacteria resemble yet not fully explored aspects of TB pathogenesis^9^. This is an approach to produce a vaccine candidate against TB entirely different from most other candidates currently in the pipeline^17^ (https://newtbvaccines.org/tb-vaccine-pipeline/preclinical-stage/). Of note, very recent evidence showing biofilm-like structures observed in TB lesions from mice, guinea pigs and humans^10^ further suggest it might be worthwhile to continue developing and conducting more in-depth characterization of BCGΔBCG1419c.

Considering the differences between first- and second-generation versions of BCGΔBCG1419c (a BCG Pasteur 1173P2 background that maintained a hygromycin resistance-Hyg^R^-gene compared with a BCG Pasteur ATCC 35734 background with no antibiotic resistance gene, respectively), we wanted to continue the non-clinical characterization of antibiotic-less BCGΔBCG1419c by determining colonial morphology and biofilm production, two phenotypes that were altered in the Hyg^R^ version of BCGΔBCG1419c compared with its parental BCG Pasteur 1173P2^5^.

Here, we found that antibiotic-less BCGΔBCG1419c maintained changes in colonial morphology and biofilm production that were restored to a full (biofilm) or partial (colony morphology) extent upon reintroduction of a single copy of the homologous gene, *Rv1357c*, under its native regulatory region, integrated into the genome of the deletion mutant (Fig. 1a,c,d); this confirms that these phenotypes are associated with the presence or absence of the c-di-GMP phosphodiesterase-encoding *BCG1419c* gene. On the other hand, by directly measuring c-di-GMP by HPLC, we confirmed that BCGΔBCG1419c produced more of this second messenger in planktonic cultures at early logarithmic growth phase (Fig. 1e), a result in agreement with our prior indirect determination of c-di-GMP levels in BCGΔBCG1419c and BCG Pasteur ATCC 35734^7^. The alterations in c-di-GMP levels might seem small in terms of the experimental value determined, yet it may represent a great scale when it comes to physiological changes. We must also bear in mind that BCGΔBCG1419c still maintains a copy of *BCG1416c* in its genome^11^. This is relevant because the Mtb homologue to *BCG1416c* is *Rv1354c*, whose enzyme has PDE activity in vitro^18^, which may contribute to a not so high increase in c-di-GMP content. Furthermore, it was shown that the recombinant product of *Rv2837c*, hydrolyzes c-di-AMP efficiently and also possesses PDE activity for c-di-GMP^19^, therefore potentially contributing to the relatively minor changes observed in our works. Taken together, along with the fact that we are not overexpressing any diguanylate cyclase-encoding gene in our work, these evidences may explain why the tightly controlled (and low level) of c-di-GMP detected in our direct HPLC measurements.

Hyg^R^ BCGΔBCG1419c lacked PDIM^5^ in biofilm cultures, yet transcriptional profiling of this same growth condition did not show significant changes in transcription of genes involved in the synthesis or export of this complex lipid, a phenotype that was not restored upon reintegration of *Rv1357c* into the mutant^5^. Perhaps not surprisingly, we found that antibiotic-less BCGΔBCG1419c was not defective in producing PDIM (Fig. 2d). This confirms that PDIM production is not dependent on the presence or absence of *BCG1419c*, at least under in vitro growth conditions, and potentially that the lack of PDIM in the hygromycin-resistant mutant was a product of an unexpected mutation.

Additional lipidomic analyses showed that, overall, this mutant had no major significant differences compared with BCG Pasteur ATCC 35734 in the amount of cardiolipin, phosphatidyl ethanol amine, phosphatidyl inositol (Fig. 2a), phosphatidyl inositol mannosides (Fig. 2b), TDM (Fig. 2c) and mycolic acids (Fig. 2e) produced in biofilm cultures, the only possible exception being a slightly increased amount of LAM, although we did not determine whether this was quantitatively significant or not (Fig. 2f).

On the other hand, when we compared the lipidomic profiles of biofilm and planktonic cultures of BCGΔBCG1419c and its parental BCG Pasteur ATCC 35734 strain, we observed two possible differences: (1) an apparent change in the production of A_c_2PIM6 and AcPIM6, although this was in a strain-independent manner, and (2) in TDM in a growth condition-independent manner. In both instances, we would need to formally evaluate the significance of these possible changes, or lack of it (Supplementary Fig. 1). Together, these results show that there were no differences in major cell envelope lipids in BCG∆BCG1419c compared with BCG Pasteur ATCC 35734 and suggest that the lack of PDIM in Hyg^R^ BCGΔBCG1419c could have been the consequence of an unanticipated spontaneous mutation that did not affect gene expression but that affected production and/or export of this complex lipid. Said hypothetical mutation could have occurred either during the mutagenesis performed to replace *BCG1419c*, or at any other moment after in vitro passages, as it is known that this can select for spontaneous mutations leading to PDIM loss^20^.

Given that most preclinical tests of novel LAV are conducted with bacteria cultured in planktonic conditions, employing shaken cultures in Middlebrook 7H9 medium with ADC/OADC supplement and detergent, in this work we decided to characterize the transcriptional profile of BCGΔBCG1419c and BCG when cultured in conditions that our group has recently employed to characterize the genome, safety, immunogenicity, efficacy, and proteome of the antibiotic-less, second-generation version of BCGΔBCG1419c^6,7,11,15^. RNASeq comparison of these strains found a few upregulated genes with most changes corresponding with downregulation of transcription in BCGΔBCG1419c compared with BCG Pasteur ATCC 35734. The main feature observed for upregulation was that BCGΔBCG1419c increased transcription of genes involved in ribosomal and/or protein synthesis, including *rrs* (16S rRNA), *rpsN1* (30S ribosomal protein), *rrl* (23S rRNA), 5S rRNA, *rpmC* (50S ribosomal protein), *glyU* (tRNA Gly), and *serT* (tRNA Ser) (Table 1).

Most changes observed in BCGΔBCG1419c were reduced transcription, including that of genes belonging to the MtrA regulon (Table 1). Among the known functions of the MtrA regulon, we recently reported that it modulates cell division and intrinsic tolerance and drug resistance^21^. Within this regulon, *pirG* (exported repetitive protein precursor, *erp*^22^) has been described as required for virulence, whereas *BCG_1539* (hypothetical invasion protein, *ripA*^23^) is required for peptidoglycan cleavage and virulence, *BCG_1540* (hypothetical invasion protein, *ripB*^24^) is also required for peptidoglycan remodeling. On the other hand, *mtc28* (secreted proline rich protein, proline rich 28 KDa antigen^25^) is an antigenic secreted protein, and *sapM* (possible acid phosphatase^26^) hydrolyzes PI3P, contributing to inhibition of phagosome-late endosome fusion (Table 1).

Moreover, we observed that transcription of several genes involved in cell wall synthesis (Table 1) were downregulated in BCGΔBCG1419c compared with BCG, among them: *murG* (*BCG_2170*, UPD-N-acetylglucosamine-N-acetylmuramyl-(pentapeptide) pyrophosphoryl-undecaprenol-N-acetylglucosamine transferase), *cswA* (*BCG_0008c*, possible membrane protein with cell wall synthesis protein CwsA domain^27^), *rfe*/*wecA* (*BCG_1362*, putative undecapaprenyl-phosphate alpha-n-acetylglucosaminyltransferase^28^), *BCG_3777* (conserved hypothetical protein with N-acetylmuramoyl-L-alanine amidase domain^29^), *aftA* (*BCG_3854*, probable conserved transmembrane protein arabinofuranosyltransferase^30^), and *BCG_3443* (conserved hypothetical protein possible triacylglycerol synthase (diacylglycerol acyltransferase) domain^31^), which are involved in synthesis of the mycolyl-arabinogalactan-peptidoglycan complex. Further to this, *manT* (*BCG_2196*, probable conserved integral membrane protein alpha(1→2) mannosyltransferase^32^), and *BCG_1059* (conserved membrane protein with dolichyl-phosphate-mannose–protein mannosyltransferase domain^33^) have been described to be required for mannose-capping of lipoarabinomannan, and protein-O-mannosylation (required for virulence), respectively.

Taken together, transcriptional data supports the notion of BCGΔBCG1419c having an altered cell wall compared with BCG, which may affect its integrity, and could explain why planktonic BCGΔBCG1419c showed constriction and concavities of the cell wall in some bacteria, while in others their cell wall was thinner than that of the parental BCG strain (Fig. 1b). On the other hand, when we compared the transcriptomic data generated in this study against proteomic data under the same experimental conditions, we found that *argF* was significantly downregulated in transcriptome (Table 1), whereas the protein was significantly upregulated in proteome comparisons between BCGΔBCG1419c and BCG^7^; *BCG_2220c* was significantly upregulated in transcriptome (Table 1), whereas the protein was significantly downregulated in proteome comparison^7^, and *BCG_0075c* was significantly downregulated both at the transcript (Table 1) and protein levels^7^. Other than this, we noticed that there was a poor correlation between transcriptomic and proteomic differences found between BCGΔBCG1419c and BCG under planktonic growth conditions (Middlebrook 7H9 media with 0.2% glycerol, 10% OADC and 0.05% Tween 80). This has already been suggested to be the case due to differences in half lives and post transcription machinery utilization^34^.

In planktonic cells, we also observed significant downregulation in BCGΔBCG1419c compared with BCG of genes belonging to different two-component systems, which are involved in response to multiple environmental cues and that are relevant for bacterial adaption. Among them, we found *BCG_3154* (*Rv3131*, putative nitroreductase) and *BCG_3153c* (*Rv3130c*, *tgs1*, triacylglycerol synthase), members of the DosR regulon, as well as reduced transcription of *narL*, and *prrA* (Table 1). Why do we think this is relevant? On the one hand, DosR and NarL have been shown to interact in vivo and co-regulate gene expression during aerobic nitrate metabolism in *M. tuberculosis*^35^. DosR is known to be induced upon hypoxia and response to NO^36^ while *prrA* also affected *M. tuberculosis* response to NO and hypoxia^37^. We also observed downregulation of genes belonging to the ArgR regulon (Table 1). Of note, in BCG, c-di-GMP was suggested to be required for adaptation to hypoxia in an ArgR-dependent manner, which induced arginine and nitrite metabolism gene clusters^38^; moreover, it is known that high c-di-GMP levels induce the expression of the DosR operon in *Mycobacterium smegmatis*^39^ to respond to oxidative stress. Together, we can hypothesize that the reduced transcription of *BCG_3154*, *BCG_3153c*, and the ArgR regulon, observed at DO600nm 0.8 in BCGΔBCG1419c compared with BCG (Table 1) might be the consequence of reduced c-di-GMP content at this stage (Fig. 1d). Whether this affects the capability of BCGΔBCG1419c to adapt to and survive under hypoxia, remains to be determined. This hypothesis coupled to the fact that PrrA and MtrA (which included several genes downregulated in BCGΔBCG1419c compared with BCG) are both targets of protein phosphorylation mediated by PknK^40^, this potentially links the activity of their target genes.

We previously found downregulation in the expression of genes involved in mycolic acids synthesis (*groEL1, fas, kasA, kasB, acpM, fabD*) in biofilm cultures of Hyg^R^ BCGΔBCG1419c compared with BCG^8^. From these genes, *fas* (Log_2_ − 0.5303, p = 0.038396) and *fabD* (Log_2_ − 0.4719, p = 0.044258) were also downregulated in planktonic cultures of BCGΔBCG1419c compared with BCG, while the remaining genes were not significantly affected (Supplementary Table 1). Downregulation of transcription of these two genes, coupled with the observed downregulation of some members of the DosR- and ArgR-regulons observed in antibiotic-less BCGΔBCG1419c compared with BCG further strengthen the notion that regardless of growth condition (biofilm or planktonic cultures), our vaccine candidate has a distinct profile to that of parental BCG. The fact that we found more differentially expressed genes in this work compared to our previous report using mature biofilm cultures^8^ may be the consequence of a greater transcriptional activity occurring in log-phase planktonic cultures used here, or because of a higher heterogeneity that may occur in biofilm cultures, which may reduce consistent transcriptional measurement in the bulk population.

Mycolic acids be incorporated into trehalose dimycolate (TDM, cord factor), which activates macrophages via Mincle and Myd88^13^. We found that macrophage activation via Mincle or Myd88 did not differ between BCG and BCGΔBCG149c, with both strains requiring these receptors to fully activate macrophages to produce TNF-α, IL-6, and G-CSF, with a more pronounced decrease in macrophages lacking Myd88 (Fig. 3 and Table 2). These findings are in agreement with the known roles of these receptors in response to mycobacterial antigens, especially TDM^13,41^, and given the absence of any significant difference in the induction of TNF-α, IL-6, and G-CSF by BCGΔBCG149c compared with BCG, this should rule out the hypothesis of potential modifications in TDM produced by BCGΔBCG149c, at least to the extent capable of inducing differential secretion of the cytokines determined here.

Taken together, our results show that BCGΔBCG1419c has a different colonial, ultrastructural, biofilm, c-di-GMP production, and transcriptomic profile (in planktonic cultures) compared with BCG, possibly explaining structural differences in their cell wall as well as suggesting potential differences that might be relevant for in vivo conditions, which deserve further explorations in future works. On the other hand, we demonstrated that there was no major change in the lipidomic profile of BCGΔBCG1419c compared with BCG, strengthening the notion that the lack of PDIM in Hyg^R^ BCGΔBCG1419c was the consequence of an unanticipated mutation. We also showed that induction of TNF-α, IL-6 or G-CSF by macrophages infected with BCGΔBCG1419c and BCG require Mincle and Myd88.

## Methods

### BCG culture

*Mycobacterium bovis* Pasteur BCG (BCG) ATCC 35734 strain and its isogenic derivative, BCGΔBCG1419c, which lacks a cyclic di-GMP phosphodiesterase encoded by the *BCG1419c* gene were grown in 7H9 liquid medium supplemented with OADC, 0.1% Tween 80 for 15 days at 37 °C and 5% CO_2_. Shaking cultures were then started at 100 rpm starting from an initial OD of 0.05 in supplemented 7H9 medium until reaching an OD of ~ 0.8. Starting from this culture, the cultures for each of the trials were prepared.

### Colonial morphology

A bacterial suspension of each of the BCG Pasteur and BCGΔBCG1419c strains was prepared at OD600nm 0.03, then two serial dilutions 1:100 were made, inoculated onto 7H10 agar plates supplemented with OADC, and 0.5% glycerol and they were incubated for 21 days at 37 °C in the presence of 5% CO_2_. After that time, to observe colonial morphology, colonies of BCG WT, BCG∆BCG1419c and complemented strains observed and recorded using a stereoscope with incident light and background illumination with a magnification of 10×.

### Ultrastructural analysis by transmission electron microscopy

Bacilli were cultured in 7H9 OADC Tween 80 medium until OD600 ≈ 1.0–1.3, harvested and fixed by immersion in a solution of 4% glutaraldehyde dissolved in cacodylate buffer for 4 h, followed by second fixation with osmium tetroxide fumes. Then, the bacterial suspension was centrifuged to form a pellet that was dehydrated with graded ethyl alcohol and embedded in Spur resin (London Resin Company, Aldermaston, UK). Sections or 70 nm to 90 nm width were obtained and placed on copper grids, contrasted with uranium salts and Reynold’s lead citrate (Electron Microscopy Sciences, Hatfield, PA, USA), and examined with an FEI Tecnai G2 Spirit Transmission Electron Microscope (Hillsboro, OR, USA).

### Biofilm culture

The BCG Pasteur and BCG∆BCG1419c strains were grown in liquid Sauton media without detergent (l-Asparagine 4 g, citric acid 2 g, KH_2_PO_4_ 0.5 g, MgSO_4_ 0.5 g, ferric ammonium citrate 0.05 g, glycerol 60 mL, 1% ZnSO_4,_ 1 mL per liter, pH adjusted to 7 with 5 N NaOH). Before this, a 20 mL pre-inoculum was prepared in Middlebrook 7H9 medium supplemented with OADC and 0.05% Tween 80 at an initial OD600nm of 0.03, cultured statically at 37 °C and 5% CO_2_, until reaching an optical density between 0.8 and 1.0. Then, cells were washed. The pellet was resuspended in 10 mL of Sauton without detergent and cultures were prepared in Sauton liquid medium without detergent at an OD600nm of 0.05, and 750 µL of total volume were placed in each well. The plates were incubated at 37 °C in the presence of 5% CO_2_ for 10 and 14 days.

### Biofilm quantification by crystal violet staining

After 10 days and 14 days of incubation, the liquid medium was removed, and all surface film and biofilm adhered to the wells remained. Immediately, 1 mL of methanol was added to each well to fix the biofilm for 5 min, the solvent was removed, and it was left to dry for 24 h at room temperature. Immediately, 1 mL of 1% crystal violet was added to each well, allowing the dye to incorporate into the biofilm for 5 min. The dye was removed, and 4 washes were carried out with distilled water to remove the residual dye. The biofilm-bound dye was extracted by adding 1 mL of 30% acetic acid to each well and allowed to incubate for 24 h. The sample from each well was diluted 1:5 in distilled water for cultures in Sauton medium and read with a UV–Vis spectrophotometer at an absorbance of 595 nm using water as a blank and the 30% acetic acid solution in the appropriate proportions according to the dilution used.

### c-di-GMP quantification by HPLC

After reaching the desired OD600nm (0.4 and 0.8), cells were washed thrice with 7H9, followed by cell lysis using the bead beating method, spin the lysed cells at 12,000 rpm for 30ʹ and recover the supernatant. The supernatant is injected into a C18 column. The mobile phase was acetonitrile (30% acetonitrile: 70% Water), and the detection wavelength was 254 nm, which is specific for cDG and not any other nucleotide. The half-life of other nucleotides differs from cDG. So, we can be sure that the molecule getting detected is cDG and not anything else. To quantify the absolute c-di-GMP concentration, we use the area under the curve (AUC) obtained for the sample by using the equation obtained from the standard curve of known concentrations. Values were normalized to the total protein concentration of each sample analyzed.

### RNA isolation

Cells in RNA later were shipped from CIATEJ to Institute for Systems Biology (ISB), at room temperature, At ISB, samples were transferred to a tube containing Lysing Matrix B (MP Biomedicals, Santa Ana, CA) and vigorously shaken at max speed for 30 s in a FastPrep 120 homogenizer (MP Biomedicals) three times. Tubes were centrifuged for 1 min (max speed), then 66 μL of 3 M sodium acetate pH 5.2 added and mixed well. Acid phenol (pH 4.2) was added at 726 μL and tubes were inverted to mix well (~ 60 times). Samples were incubated at 65 °C for 5 min, inverting tubes to mix samples every 30 s. Then, centrifuged at 14,000 rpm for 5 min and upper aqueous phase was transferred to a new tube. 3 M sodium acetate (pH 5.2) was added at 1/10th volume along with 3× volumes of 100% ethanol. Sample was mixed well and incubated at − 20 °C for 1 h or overnight. Following incubation, samples were centrifuged at 14,000 rpm for 30 min at 4 °C, ethanol was discarded and 500 μL of 70% ethanol was added. Samples were centrifuged again at 14,000 rpm for 10 min at 4 °C, supernatant discarded, and any residual ethanol removed using pipet. Pellet was allowed to air dry, resuspended in 30–40 μL of RNase free water and quantified by Nanodrop (Thermo Scientific). This was followed by in solution genomic DNA digestion using RQ1 Dnase (Promega) following manufacturer’s recommendation. RNA quality was analyzed in a 2100 Bioanalyzer system (Agilent Technologies). Total RNA samples were depleted of ribosomal RNA using the Ribo-Zero Bacteria rRNA Removal Kit (Illumina, San Diego, CA). Samples were prepared with TrueSeq Stranded mRNA HT library preparation kit (Illumina, San Diego, CA) and sequenced on the NextSeq 2000 instrument with NextSeq P2 (200 cycles) kit.

### RNA-seq analysis

Raw FASTQ read data were processed using the R package DuffyNGS as described previously^42^. Briefly, raw reads were filtered for rRNA transcripts and aligned against the *M. bovis* BCG str. Pasteur (1173P2) genome with Bowtie2^43^, using the command line option “very-sensitive.” BAM files recorded both uniquely mapped and multiply mapped reads to each of the forward and reverse strands of the genome(s) at single-nucleotide resolution. Gene transcript abundance was then measured by summing total reads landing inside annotated gene boundaries, expressed as both RPKM and raw read counts. Two stringencies of gene abundance were provided using all aligned reads and by just counting uniquely aligned reads. The raw and processed RNA-seq data generated for this study are available in the Gene Expression Omnibus under accession number GSE251760.

### Differentially expressed genes

A panel of 5 DE tools was used to identify gene expression changes between the mutant Δ1419 and wild type. The tools included (i) RoundRobin (in-house); (ii) RankProduct^44^; (iii) significance analysis of microarrays (SAM)^45^; (iv) EdgeR^46^; and (v) DESeq2^47^. Each DE tool was called with appropriate default parameters and operated on the same set of transcription results, using RPKM abundance units for RoundRobin, RankProduct, and SAM and raw read count abundance units for DESeq2 and EdgeR. All 5 DE results were then synthesized, by combining gene DE rank positions across all 5 DE tools. Specifically, a gene’s rank position in all 5 results was averaged, using a generalized mean to the 1/2 power, to yield the gene’s final net rank position. Each DE tool’s explicit measurements of differential expression (fold change) and significance (p-value) were similarly combined via appropriate averaging (arithmetic and geometric mean, respectively). Genes with *P*-value (averaged *P*-value across the tools) below 0.05 were considered differentially expressed. We performed BLAST^48^ to identify homologs in *M. tuberculosis* H37Rv and functional term clusters were defined by DAVID^16^ for either up or down-regulated significantly expressed genes. Conserved domains were searched for proteins with Conserved Hypothetical Functions at https://www.ncbi.nlm.nih.gov/Structure/cdd/wrpsb.cgi with default parameters.

### Lipid extraction and analyses

Planktonic and biofilm cultures produced at CIATEJ were lyophilized and shipped at room temperature to University of Massachusetts at Amherst (UMA). There, to harvest phospholipids and glycolipids, 10 volumes (v/w) of chloroform/methanol (2:1, v/v) were added to lyophilized cells. The cell suspension was briefly vortexed, sonicated, and incubated at room temperature for at least one-hour. The suspension was spun down at 16,900×*g* on a microfuge for 1 min to harvest the supernatant. We repeated the extraction with10 volumes of chloroform/methanol (2:1, v/v) and 10 volumes of chloroform/methanol/water (1:2:0.8, v/v/v) against the same pellet. The combined lipid extract was further purified by n-butanol/water phase partitioning as previously^49^. The final lipid extract was analyzed by high-performance thin layer chromatography (HPTLC) (silica gel 60, EMD Merck) using the solvent systems as follows: phospholipids were developed on an HPTLC plate in a solvent containing chloroform/methanol/13 M ammonia/1 M ammonium acetate/water (180:140:9:9:23, v/v/v/v/v) and detected by molybdenum blue staining. PIMs were developed on an HPTLC plate in a solvent containing chloroform/methanol/13 M ammonia/1 M ammonium acetate/water (180:140:9:9:23, v/v/v/v/v) and detected by orcinol staining. TDM was developed on an HPTLC plate in a solvent containing chloroform/methanol/water (90:10:1, v/v/v) and detected by orcinol staining.

The delipidated cell pellet after the lipid extraction described above was subjected to LM/LAM extraction using hot phenol and analyzed by SDS-PAGE (15% gel) and visualized by Pro-Q Emerald 488 Glycoprotein Gel and Blot Stain Kit (Thermo Fisher) as described previously^50^.

For the analysis of PDIMs, we suspended 100 mg of lyophilized cells in 1 ml of methanol/0.3% (w/v) aqueous sodium chloride (10:1) and 0.5 ml of petroleum ether. The cell suspension was vortexed for 15 min to emulsify the two layers, and the top petroleum ether phase was harvested after brief centrifugation. The extraction was repeated with another 0.5 ml of petroleum ether against the same methanol/water phase. The combined petroleum ether phase was dried, resuspended in 100 µl of petroleum ether, and 10 µl was spotted on an HPTLC plate. HPTLC plate was developed in petroleum ether/diethyl ether (9:1) once, and PDIMs plus TAGs were visualized by phosphomolybdic acid staining.

For mycolic acid methyl ester (MAME) and fatty acid methyl ester (FAME) analysis, 50 mg of lyophilized cells were incubated overnight at 100 °C in 2 ml of 15% tetra butyl ammonium hydroxide. The alkaline-hydrolyzed materials were mixed with 2 ml water, 1 ml dichloromethane, and 250 µl iodomethane to produce methyl esters of fatty acids and mycolic acids. The mixture was incubated at room temperature for 30 min with mixing. The upper aqueous layer was discarded, and the lower organic layer was washed with 3 ml of 1 M HCl and 3 ml of water. The organic layer was dried and resuspended in 500 µl of toluene/acetonitrile (2:3). The MAMEs and FAMEs were separated by HPTLC with petroleum ether/acetone (95:5) once and visualized by phosphomolybdic acid staining.

### Macrophage obtention and determination of cytokines

In each experiment, bone-marrow derived macrophages (BMM) were differentiated from 2 different mice for each genotype (C57BL/6, Mincle-KO (MNA), and Myd88-KO mice in complete DMEM containing 10% L-cell conditioned media as a source of M-CSF for 7 days. BMM were harvested using Accutase (Sigma-Merck), washed and plated in cDMEM without M-CSF or antibiotics in 96 flat-bottom well plates at 2 × 10^5^ cells per well. After overnight incubation, BCG strains were added at MOI 5. 24 h later, the supernatants were harvested and analyzed by ELISA (DuoSet, R&D Systems) for TNF-α, IL-6 and G-CSF according to the manufacturer’s instructions. BMM were plated for stimulation in duplicate or triplicate wells, giving us average cytokine values from 4 to 6 wells in each experiment.

### Supplementary Information

Supplementary Figure 1.

Supplementary Legends.

Supplementary Table 1.
