# Supplementary figures and images for: Comparison of the transcriptome, lipidome, and c-di-GMP production between BCGΔBCG1419c and BCG, with Mincle- and Myd88-dependent induction of proinflammatory cytokines in murine macrophages
Source: Sci Rep. 2024 May 24;14:11898. doi: 10.1038/s41598-024-61815-8 (PMC11126594; doi:10.1038/s41598-024-61815-8)

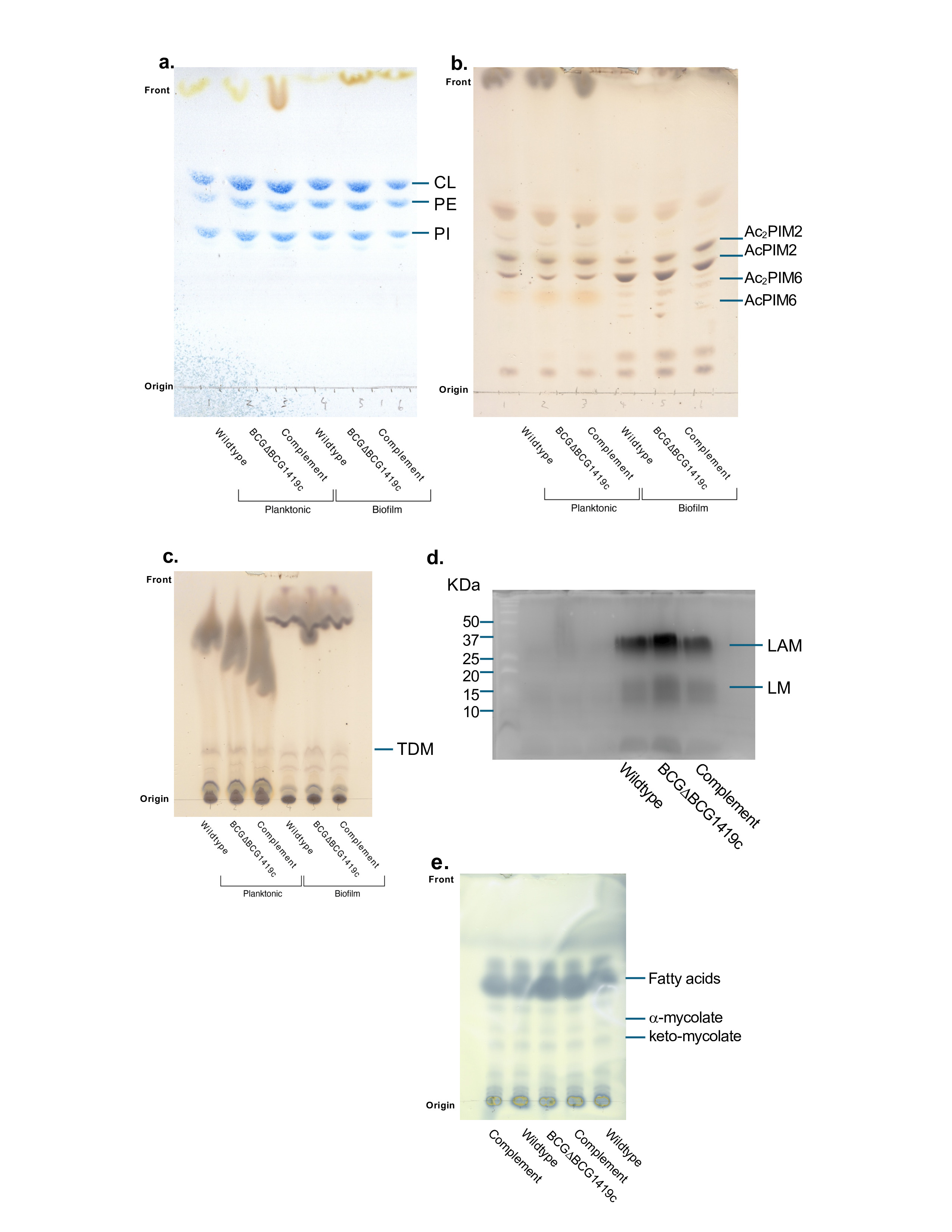

Supplement: Supplementary file 1 — Supplementary Figure 1. [file 41598_2024_61815_MOESM1_ESM.jpg]
