# Supplementary material for: Comparison of the transcriptome, lipidome, and c-di-GMP production between BCGΔBCG1419c and BCG, with Mincle- and Myd88-dependent induction of proinflammatory cytokines in murine macrophages
Source: Sci Rep. 2024 May 24;14:11898. doi: 10.1038/s41598-024-61815-8 (PMC11126594; doi:10.1038/s41598-024-61815-8)
Supplement: Supplementary file 2 — Supplementary Legends. [file 41598_2024_61815_MOESM2_ESM.docx]

Supplementary Figure 1. Analysis of cell envelope lipids in biofilm versus planktonic cultures. The lipids of the different BCG strains sampled from planktonic cultures in 7H9 OADC 0.05% Tween 80 at OD600nm or 2 weeks-old biofilm cultures were extracted and analyzed by HPTLC as described in Methods. (a). Phospholipids, including cardiolipin (CL), phosphatidylethanolamine (PE), and phosphatidylinositol (PI). (b) Phosphatidyl-myo-inositol Mannosides (PIMs). (c) Trehalose dimycolates (TDM). (d) Mannose-based lipoglycans such as lipomannan (LM) and lipoarabinomannan (LAM). (e) Fatty acids and mycolic acids methyl ester derivatives (α- and keto-mycolic acids). Analyses was performed with triplicate samples and a representative image is shown for each lipid class.
